# Incidence of SARS-CoV-2 Infection and Factors Associated With Complete COVID-19 Vaccine Uptake Among Migrant Origin Persons in Finland

**DOI:** 10.3389/ijph.2023.1605547

**Published:** 2023-05-03

**Authors:** Kalpana Bastola, Hanna Nohynek, Eero Lilja, Anu E. Castaneda, Sara Austero, Hannamaria Kuusio, Natalia Skogberg

**Affiliations:** National Institute for Health and Welfare, Helsinki, Finland

**Keywords:** COVID-19, sociodemographic factors, migrant, vaccine uptake, SARS-CoV-2 infection

## Abstract

**Objective:** We examined incidence of SARS-CoV-2 infection, COVID-19 vaccine uptake and factors associated with complete COVID-19 vaccine uptake among persons of migrant origin in Finland.

**Methods:** Data on laboratory-confirmed SARS-CoV-2 infection and COVID-19 vaccine doses between March 2020 and November 2021 were linked to FinMonik register sample (*n* = 13,223) and MigCOVID (*n* = 3,668) survey data using unique personal identifier. Logistic regression was the main method of analyses.

**Results:** Among FinMonik sample, complete COVID-19 vaccine uptake was lower among persons of Russia/former Soviet Union, Estonia, and rest of Africa and higher among persons of Southeast Asia, rest of Asia, and the Middle East/North Africa than among persons originating from Europe/North America/Oceania. Male sex, younger age, migration age (<18 years) and shorter length of residence were associated with lower vaccine uptake among FinMonik sample, whereas younger age, being economically inactive, poorer language skills, experiences of discrimination and psychological distress were associated with lower vaccine uptake among MigCOVID sub-sample.

**Conclusion:** Our Findings point to a further need of tailored and targeted communication and community outreach strategies to increase vaccine uptake among persons of migrant origin.

## Introduction

The corona virus disease (COVID-19) pandemic and related restrictive measures have severely affected societies across the globe, especially the vulnerable communities, including persons of migrant origin and/or of diverse ethnic background (1, 2). High vaccine uptake is crucial to reduce COVID-19-related hospitalizations and deaths. The World Health Organization (WHO) has classified disadvantaged sociodemographic subpopulations as one of the high-risk groups for SARS-CoV-2 infection and a high priority group for COVID-19 vaccination (3). Persons of migrant origin often belong to less advantaged sociodemographic subpopulations in high-income countries and have been reported to have a higher risk for SARS-CoV-2 infection compared with the general population (4).

Differences in the incidence of SARS-CoV-2 infection have also been reported by migrant origin and ethnicity compared to the general population in high-income countries (4, 5). In a systematic review, the high-risk groups for SARS-CoV-2 infection were identified based on geographical region of origin (Asian, Southeast Asian, and African origin), and by ethnicity (Black American, Hispanic, and Latino origin), compared to the general population in the same countries (5). Differences in the incidence of SARS-CoV-2 infection have also been reported by migrant status, with a higher risk for SARS-CoV-2 infection among undocumented migrants, migrant healthcare workers, and persons living in refugee camps (4).

Emerging evidence from high-income countries shows differences in COVID-19 vaccine uptake by migrant origin and ethnicity (6–8). A recent study from Norway reported lower vaccine uptake among migrant origin persons (either the person themselves or their parents born outside of Norway) than Norwegian born persons with Norwegian born parents (8). Vaccine uptake also varied by country of birth in Norway, from around 45% among persons born in Latvia, Bulgaria, Poland, Romania and Lithuania to 92% for persons born in Vietnam, Thailand and Sri Lanka (9). Another study from the United Kingdom (UK) showed that persons of Black (74%), Asian (85%), mixed (82.5%) and other (76%) ethnicity had lower vaccine uptake than the general population in Wales (94%) (10).

Thus, even though there is some evidence in support of differences in SARS-CoV-2 infection and vaccine uptake incidence across different population groups, generalizability and cross-country comparison of these findings across studies is somewhat challenging due to large variations in how the study groups have been formulated (i.e., specific country of birth, geographical region of origin, and ethnicity). When examining vaccine uptake, it is important to consider not only the number of vaccine doses, but also whether the same person has also had a SARS-CoV-2 infection. This is because the official recommendations by the Finnish health authorities considered a previous SARS-CoV-2 infection as equivalent to one dose of vaccine (11, 12). Following the official recommendations, full vaccine coverage was considered as two doses of COVID-19 vaccine or one dose of COVID-19 and a previous SARS-CoV-2 infection (13). Considering the high prevalence of SARS-CoV-2 infection among migrant origin populations, it is particularly important to take into account both previous infection and the number of vaccine doses to get an accurate estimate of those who had complete vaccine uptake based on the official recommendations.

The main reasons identified behind the greater risk for SARS-CoV-2 infection among migrant origin populations were high-risk occupations, overcrowded housing and barriers to healthcare, including inadequate information, language barriers, and restricted entitlement to services (4). Similarly, pre-existing mistrust of formal services, lack of information about the vaccine’s safety, misinformation, inaccessible communications, and logistical issues were common barriers among the migrant population toward COVID-19 vaccination uptake in the UK (14). It has also been suggested that these adverse outcomes among migrant groups may be the result of a complex interaction of socioeconomic disadvantages and underlying health status (15).

In Finland, at the end of 2020, almost 8% of the population was of migrant origin (16). Incidence of SARS-CoV-2 infection and vaccine uptake have not been systematically reported in Finland by country or region of origin or mother tongue. However, some reports have been published on higher incidence of SARS-CoV-2 infection among persons speaking other than official languages in Finland (17, 18). Persons speaking other than official languages have also been reported to have lower vaccine uptake (45%) compared with the general population (74%) by beginning of October 2021 (19). Therefore, this study was initiated to provide critical information for a more detailed understanding of the phenomenon, as well as to aid the planning and promotion of COVID-19 vaccine uptake among diverse population groups. This study aimed to examine the incidence of SARS-CoV-2 infection, COVID-19 vaccine uptake, as well as the association of sociodemographic and health-related factors with COVID-19 vaccine uptake among persons of migrant origin in Finland.

## Methods

### Study Population and Data Source

The baseline data of our study is based on the Survey on Wellbeing among Foreign-Born Population (FinMonik) study which provided information on sociodemographic characteristics of the study population. Information on the COVID-19 vaccination status and the laboratory confirmed SAR-COV-2 findings were obtained from the Finnish Vaccine Register and Infectious Disease Register. Data linkage across registers was possible based on the unique personal identity number. The method of data analyses that we used are generally used in this type of register-based studies.

**FIGURE 1:**
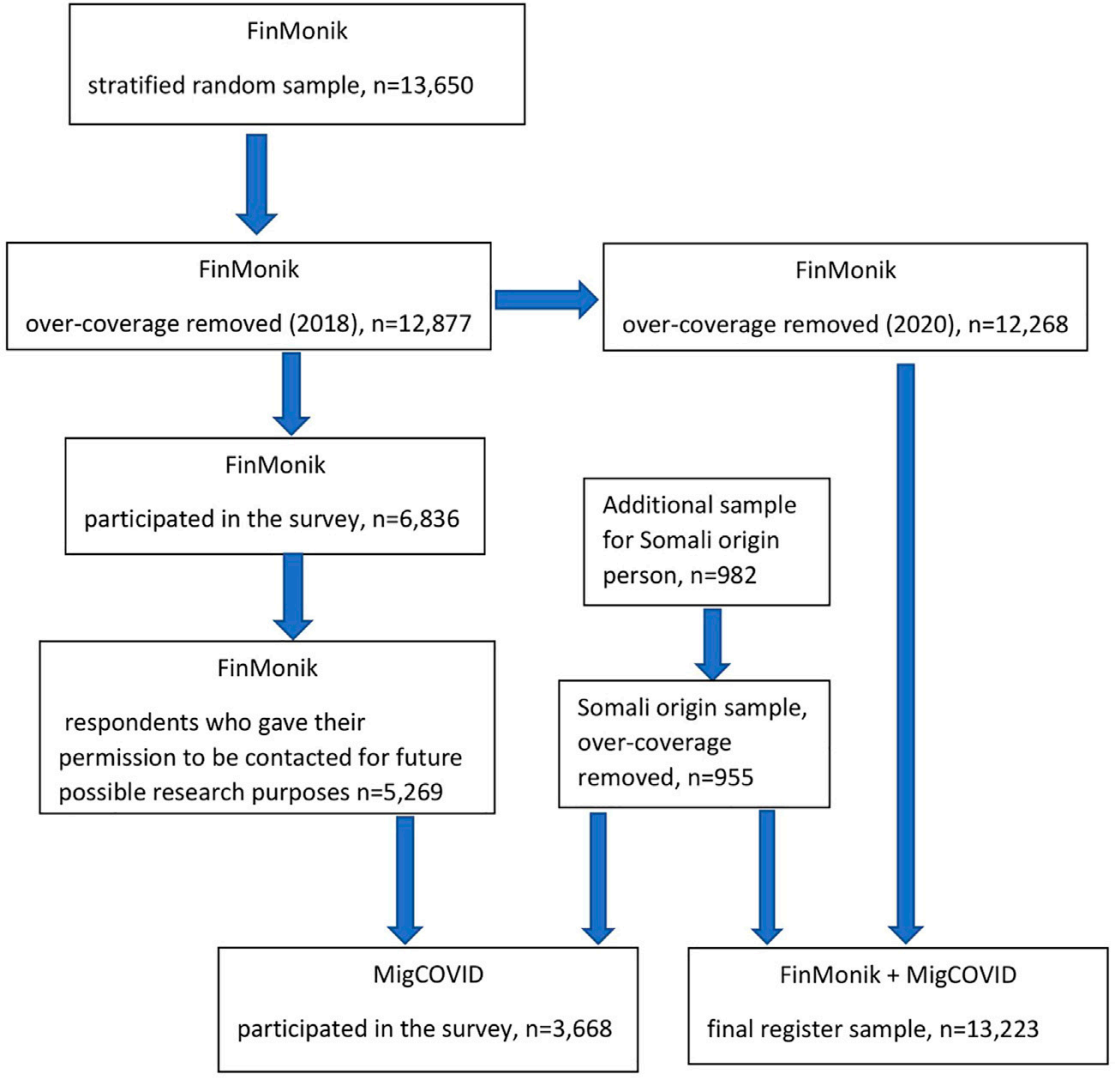
Flowchart of study population (Finland, 2022).

The study sample is presented in a flowchart (Figure 1). This study is based on a stratified randomly drawn total register FinMonik sample (20), originally drawn from the Finnish Population Register in 2018 (*n* = 12,877) and updated in 2020. The update led to exclusion of *n* = 609 persons identified as overcoverage, i.e., they no longer lived in Finland or had died. Additionally, a supplementary sample of Somalia-born persons (*n* = 955) drawn in 2020 by the Finnish Institute for Health and Welfare’s (THL). The combined total register sample in this study therefore constituted of *n* = 13,223 persons. The selection criteria were age 18–64 years, country of birth other than Finland, parents’ countries of birth other than Finland, the participant not adopted to Finland, length of residence in Finland for at least 1 year and currently living in mainland Finland. The sociodemographic data was available for the study population from the Finnish Population Register. This data was supplemented with register data from the Finnish Vaccine Register and Infectious Disease Register (COVID-19 vaccination status and the laboratory confirmed SAR-COV-2 findings). Data linkage was possible based on the unique personal identification number, which everyone residing in Finland is given.

Persons belonging to the FinMonik total register sample were invited to take part in the FinMonik Survey conducted between 2018 and 2019 and described in more detail elsewhere (20). Altogether *n* = 6,836 persons (53%) took part in the FinMonik Survey. These same persons, excluding those who declined from further contact, had moved away from Finland or had died by 2020 (*n* = 1,567), were invited to participate in a follow-up survey in 2020. This follow-up survey was called the Impact of Coronavirus Epidemic on Wellbeing among Foreign-born Population (MigCOVID) Survey and was conducted between October 2020 and February 2021 (2). Persons belonging to the Somali-born supplementary sample drawn in 2020 were also invited to take part in the MigCOVID Survey. Data were collected with an electronic or paper-based questionnaire that was available in 18 different languages. These were supplemented with multilingual telephone interviews conducted by trained fieldwork personnel. In total, 3,668 persons took part in the MigCOVID Survey, with a participation rate of 60% out of those invited to participate.

### Study Variables

Migrant groups were formed based on the United Nations Standard country or area codes for statistical use (21). However, a slight deviation was made from the United Nations area codes by including Iran and Afghanistan in the Middle East and North Africa group due to the cultural similarities. For this analysis, participants were classified into seven regional categories: 1) Russia and the former Soviet Union; 2) Estonia; 3) Europe (excl. Russia and Estonia), North America, Oceania; 4) the Middle East and North Africa; 5) Africa (excl. North Africa); 6) Southeast Asia and 7) Asia (excl. Southeast Asia) and Latin America. The list of the countries in each regional group is available in the Supplementary Table S1.

Our composite outcome variable, complete COVID-19 vaccination uptake, was based on two variables: incidence of SARS-CoV-2 infection and the number of COVID-19 vaccine doses. We have included previous COVID-19 infection as a part of vaccine uptake because a previous SARS-CoV-2 infection can be considered equivalent to one dose of vaccine in protecting from future COVID-19 infection (11, 12). We defined vaccine uptake as complete if the person had a previous laboratory-confirmed SARS-CoV-2 infection and at least one vaccine dose or if a person had two or more vaccine doses. The vaccination uptake was considered incomplete if a person had no previous laboratory-confirmed SARS-CoV-2 infection and less than two vaccine doses or previous SARS-CoV-2 infection and zero vaccine doses. Finally, the outcome variable “complete vaccine uptake” was categorized as yes/no.

Information on age, sex, municipality type and date of first registration of residence in Finland were obtained from the Finnish Population Register. Age was categorized into five age strata: 20–29, 30–39, 40–49, 50–59 and 60–66 years. Sex was categorized as male or female. The municipality type was categorized as urban, semi-urban and rural. Length of residence was calculated based on the first date of registration of residence in Finland and on the date when the survey sample was drawn/updated and was categorized as less than 7 years, 7–11 years, and 12 years or more. Age at migration was calculated based on the first date of registration in Finland and the age of the person at the point when the sample was drawn/updated and dichotomized as below 18 years and 18 years or older.

Information on the level of education, economic activity, Finnish or Swedish language proficiency, self-rated health, psychological distress, and experiences of discrimination was obtained from the MigCOVID Survey. Level of education was categorized as basic level or less, secondary level, and higher level (university or higher). Economic activity was defined as working full-time/part-time, student, unemployed, or other. Proficiency in the Finnish or Swedish language (official languages of Finland) was defined as beginner/intermediate or excellent. Self-rated health was dichotomized as average/fairly poor/poor, fairly good/good. Psychological distress was measured with the Mental Health Index (MHI-5) (22), dichotomized as no psychological distress (if the cumulative point were >52), psychological distress (if the cumulative point were<=52). Perceived discrimination was categorized as yes or no. Experiences of discrimination were assessed by asking the participants whether during the COVID-19 epidemic they have been: 1) treated with less respect than others; 2) called names or verbally insulted; 3) threatened or harassed. A joint variable was created including those who reported yes to at least one of these categories and finally categorized as yes or no.

### Statistical Analyses

All statistical analyses were performed with Stata version 17 (Stata Corp LP, College Station, TX, USA) and SAS 9.4/SUDAAN 11.0.3. Inverse sampling probability weights were applied in all analyses to correct for the effect of differential sampling probabilities and to reduce the effect of non-response bias. The calculation of the weights has been described elsewhere (2). Logistic regression model was the main method of analysis and odds ratio (OR) and 95% confidence interval were reported (CI) as the measure of association. Model I was crude model, Model II was adjusted for age, sex, age at migration, length of stay in Finland, and Model III was the full model including all variables presented in the table. Persons originating from Europe (excl. Russia, Estonia)/North America/Oceania were the reference group in the regression models.

## Results

Table 1 presents the background characteristics of the study population. Among our study participants, the largest group was of Russian/former Soviet Union origin while the smallest group was of Southeast Asian origin. The largest group of participants belonged to the 30–39 years age category across all regional groups. The proportion of female participants was largest in the Southeast Asian origin group, whereas it was least in the Middle Eastern and North African origin group. Most of the participants in all regional groups lived in an urban area and had moved to Finland after 18 years of age. More than 70.0% of the participants had been living in Finland for 7 years or more. Among the MigCOVID sub-sample, the highest level of education varied from 24.8% among those of Estonian to 61.6% among those of Asian (excl. Southeast Asia)/Latin American origin. The proportion of those working either full-time or part-time varied from 46.1% among persons of Middle Eastern/North African origin to 73.1% among persons of Southeast Asian origin. Only about 20.0% of persons of Asian/Latin American origin had excellent Finnish/Swedish language skills. Among those who had experienced discrimination, the largest proportion was among persons of Middle Eastern/North African origin, followed by persons of Southeast Asian and African (Excl. North Africa) origin. The proportion of those reporting fairly good/good health ranged from 66.5% to 81.0% depending on the regional group.

**TABLE 1:** Background characteristics of the total study sample by region of origin, (Finland, 2022).

Variables	Russia/former Soviet Union, (*n* = 2,875)	Estonia, (*n* = 1,806)	Europe (excl. Russia, Estonia)/North America/Oceania, (*n* = 2,609)	Middle East/North Africa, (*n* = 2,066)	Africa (excl. North Africa), (*n* = 1,211)	Southeast Asia, (*n* = 1,079)	Asia (excl. Southeast Asia)/Latin America, (*n* = 1,577)	Total N = 13,223
	n (%)	n (%)	n (%)	n (%)	n (%)	n (%)	n (%)	n (%)
Total study population (N = 13,223)								
Age								
20–29	383 (13.3)	278 (15.4)	344 (13.2)	482 (23.3)	383 (31.6)	258 (23.9)	301 (19.1)	2,429 (18.4)
30–39	787 (27.4)	504 (27.9)	925 (35.0)	765 (37.0)	513 (42.4)	339 (31.4)	669 (42.5)	4,502 (34.0)
40–49	719 (25.0)	474 (26.3)	726 (27.9)	452 (21.9)	213 (17.7)	285 (26.4)	353 (22.4)	3,223 (24.4)
50–59	620 (21.6)	395 (21.9)	431 (16.5)	280 (13.6)	84 (6.9)	158 (14.7)	208 (13.2)	2,176 (16.5)
60–66	366 (12.7)	154 (8.6)	183 (7.0)	87 (4.2)	18 (1.5)	38 (3.6)	46 (2.9)	893 (6.8)
Sex								
Male	1,209 (42.0)	895 (49.6)	1,584 (60.7)	1,392 (67.4)	680 (56.2)	287 (26.6)	824 (52.2)	6,871 (52.0)
Female	1,666 (58.0)	911 (50.5)	1,024 (39.3)	674 (32.6)	531 (43.8)	792 (73.4)	753 (47.8)	6,352 (48.0)
Municipality type								
Urban	2,464 (85.7)	1,543 (85.5)	2,240 (85.9)	1,950 (94.4)	1,179 (97.3)	921 (85.4)	1,515 (96.1)	11,812 (89.3)
Semi-urban	211 (7.4)	166 (9.2)	206 (7.9)	93 (4.5)	25 (2.1)	79 (7.3)	43 (2.7)	823 (6.2)
Rural	199 (6.9)	97 (5.4)	163 (6.3)	23 (1.1)	8 (0.6)	80 (7.4)	19 (1.2)	588 (4.5)
Age at migration								
less than 18 years	657 (22.8)	238 (13.2)	255 (9.8)	356 (17.2)	497 (41.1)	168 (15.5)	129 (8.2)	2,300 (17.4)
18 years or more	2,218 (77.2)	1,569 (86.8)	2,354 (90.2)	1,710 (82.8)	714 (58.9)	912 (84.5)	1,447 (91.8)	10,923 (82.6)
Length of stay in Finland, years								
Less than 7 years	311 (10.8)	225 (12.5)	560 (21.5)	655 (31.7)	225 (18.6)	295 (27.4)	445 (28.3)	2,716 (20.5)
7–11 years	573 (19.9)	773 (42.8)	671 (25.7)	517 (25.0)	320 (26.4)	303 (28.1)	493 (31.3)	3,650 (27.6)
12 years or more	1,991 (69.3)	808 (44.7)	1,379 (52.8)	894 (43.3)	667 (55.0)	481 (44.5)	639 (40.5)	6,857 (51.9)
**MigCOVID Survey**	(***n* = 797)**	**(*n* = 498)**	**(*n* = 691)**	**(*n* = 572)**	**(*n* = 335)**	**(*n* = 289)**	**(*n* = 487)**	**(*n* = 3,668)**
subsample (*n* = 3,668)								
Level of education								
Basic level or less	48 (6.1)	78 (16.0)	81 (11.8)	103 (18.5)	67 (20.9)	69 (24.4)	38 (7.9)	484 (13.5)
Secondary level	443 (56.6)	291 (59.2)	229 (33.7)	280 (50.2)	142 (44.3)	136 (47.9)	148 (30.6)	1,667 (46.3)
Higher level	292 (37.3)	122 (24.8)	371 (54.5)	174 (31.3)	111 (34.8)	79 (27.8)	299 (61.6)	1,447 (40.2)
Economic activity								
Working full-time/part-time	503 (64.5)	438 (70.7)	488 (72.3)	256 (46.1)	199 (60.8)	207 (73.1)	315 (66.2)	2,315 (64.5)
Student	45 (5.7)	20 (4.0)	45 (6.7)	119 (21.4)	59 (18.1)	16 (5.5)	63 (13.3)	366 (10.2)
Unemployed	133 (17.0)	71 (14.5)	61 (9.1)	97 (17.4)	36 (10.9)	40 (14.0)	68 (14.2)	505 (14.1)
Other	100 (12.8)	53 (10.8)	80 (11.9)	84 (15.1)	33 (10.2)	21 (7.3)	30 (6.3)	401 (11.2)
Finnish/Swedish language skills								
Beginner/intermediate	456 (55.9)	172 (45.1)	500 (63.7)	383 (72.2)	109 (49.4)	234 (78.6)	446 (80.5)	2,300 (64.2)
Excellent	360 (44.1)	209 (54.9)	285 (36.3)	146 (27.6)	111 (50.6)	64 (21.4)	108 (19.5)	1,284 (35.8)
MHI index								
No psychological distress (points >52)	661 (85.2)	386 (79.7)	510 (76.3)	384 (70.5)	271 (86.0)	243 (85.6)	383 (82.8)	2,838 (80.3)
Psychological distress (points<=52)	115 (14.8)	98 (20.3)	158 (23.7)	161 (29.5)	44 (14.0)	41 (14.4)	80 (17.2)	698 (19.7)
Perceived discrimination								
No	743 (93.7)	448 (90.1)	604 (88.5)	432 (76.0)	259 (77.9)	223 (77.3)	387 (80.5)	3,095 (85.0)
Yes	50 (6.3)	49 (9.9)	79 (11.5)	137 (24.0)	74 (22.10)	66 (23.0)	94 (19.6)	548 (15.0)
Self-rated health								
Average/fairly poor/poor	216 (27.2)	132 (26.6)	191 (28.1)	170 (29.7)	63 (19.0)	94 (32.4)	162 (33.5)	1,028 (28.2)
Fairly good/good	577 (72.8)	365 (73.4)	491 (72.0)	402 (70.3)	268 (81.0)	195 (67.6)	323 (66.5)	2,621 (71.8)

Table 2 presents the incidence of laboratory-confirmed SARS-CoV-2 infection and COVID-19 vaccine uptake among migrant origin persons among total sample. The highest incidence of SARS-CoV-2 infection was observed among persons originating from the African (excl. North Africa) region (19.4%) and the Middle Eastern/North African region (19.2%) respectively. The lowest incidence of laboratory-confirmed SARS-CoV-2 was observed among persons of Southeast Asian origin (5.0%). The proportion of those who have received two doses of COVID-19 vaccines was lowest among persons of Estonian origin (38.8%) and highest among persons of Southeast Asian origin (80.6%). Finally, the complete vaccination uptake ranged from 85.0% among Southeast Asians to 41.0% among Estonians. The incidence of laboratory-confirmed SARS-CoV-2 infection and COVID-19 vaccine uptake among the MigCOVID sub-sample are presented as a Supplementary Table S2.

**TABLE 2:** Incidence of laboratory confirmed SARS-CoV-2 infection and COVID-19 vaccine uptake in the total study sample, (Finland, 2022).

Migrant groups	SARS-CoV-2 infection	Number of COVID-19 vaccine doses				Complete vaccine uptake^a^
		0	1	2	3	
	% (95% CI)	% (95% CI)	% (95% CI)	% (95% CI)	% (95% CI)	% (95% CI)
Russia/former Soviet Union (*n* = 2,875)	7.1 (6–8.5)	38.7 (36.5–41.0)	6.7 (5.6–7.8)	53.1 (50.8–55.4)	1.5 (1.1–2.1)	55.6 (53.4–57.9)
Estonia (*n* = 1,806)	9.4 (7.5–11.7)	54.5 (51.1–57.8)	5.1 (3.8–6.9)	38.8 (35.6–42.2)	1.6 (1.0–2.3)	41.2 (38–44.6)
Europe (excl. Russia, Estonia)/North America/Oceania (*n* = 2,609)	7.1 (5.8–8.7)	31.8 (29.3–34.4)	5.4 (4.2–6.8)	61.6 (58.9–64.2)	1.3 (0.8–1.9)	63.6 (60.9–66.2)
Middle East/North Africa (*n* = 2,066)	19.2 (16.7–22.0)	25 (22.4–27.8)	9.9 (8.1–12.0)	63.4 (60.3–66.3)	1.7 (1–2.8)	69.1 (66.1–71.9)
Africa (excl. North Africa) (*n* = 1,211)	19.4 (16.8–22.3)	43.2 (39.6–46.9)	10.9 (9.0–13.2)	43.8 (40.2–47.6)	2.0 (1.1–3.6)	48.7 (45–52.4)
Southeast Asia (*n* = 1,079)	6.8 (4.8–9.5)	10.8 (8.6–13.5)	5.8 (4.1–8.3)	80.6 (77.2–83.7)	2.7 (1.7–4.4)	84.6 (81.4–87.3)
Asia (excl. Southeast Asia)/Latin America (*n* = 1,577)	5.1 (3.5–7.2)	22.4 (19.4–25.7)	3.0 (2.0–4.4)	72.3 (68.8–75.5)	2.3 (1.5–3.7)	75.5 (72.1–78.6)
Total (*n* = 13,223)	10.2 (9.4–11.0)	33.6 (32.5–34.7)	6.6 (6.0–7.2)	58.1 (56.9–59.3)	1.7 (1.5–2.1)	61.4 (60.3–62.6)

^a^ Complete vaccine uptake is defined as complete, if a person had a previous SARS-Cov-2 infection and one dose vaccination or if person has two or more vaccination dose and no previous SARS-Cov-2 infection.

Table 3 presents the unadjusted and adjusted odds ratio (OR) and 95% confidence interval (CI) for the association of sociodemographic and health-related factors with complete vaccine uptake in each migrant origin group compared with the Europe (excl. Russia and Estonia)/North America/Oceania group among total study sample. In both unadjusted (Model I) and adjusted model (Model II), the results were similar. Persons of Southeast Asian, Asian (excl. Southeast Asia)/Latin American and the Middle Eastern/North African origin were more likely to have complete vaccine uptake whereas, persons of Russia/former Soviet Union, Estonian and African (excl. North Africa) origin were less likely to have complete vaccine uptake compared with persons of European (excl. Estonian)/North American/Oceanian origin. Compared with those in age-group 20–34, those in age groups 35–49 and 50–66 age groups were more likely to have complete vaccine uptake. Those who migrated after 18 years of age were more likely to have complete vaccine uptake compared with those who migrated before the age of 18 years.

**TABLE 3:** The association of sociodemographic and health-related factors with complete COVID-19 vaccine uptake in the total study sample, Odds Ratio (OR) and 95% confidence interval (CI), (Finland, 2022).

Region of origin	Model I (*n* = 13,223)		Model II (*n* = 13,223)	
	OR (95% CI)	*p*-value	OR (95% CI)	*p*-value
Europe (excl. Russia, Estonia)/North America/Oceania	Reference		Reference	
Russia/former Soviet Union	**0.71 (0.64–0.80)**	**<0.001**	**0.68 (0.61–0.76)**	**<0.001**
Estonia	**0.40 (0.36–0.45)**	**<0.001**	**0.40 (0.36–0.46)**	**<0.001**
Middle East/North Africa	**1.28 (1.13–1.44)**	**<0.001**	**1.41 (1.25–1.60)**	**<0.001**
Africa (excl. North Africa)	**0.54 (0.47–0.62)**	**<0.001**	**0.63 (0.54–0.72)**	**<0.001**
Southeast Asia	**3.14 (2.62–3.78)**	**<0.001**	**3.34 (2.77–4.02)**	**<0.001**
Asia (excl. Southeast Asia)/Latin America	**1.76 (1.53–2.02)**	**<0.001**	**1.90 (1.65–2.19)**	**<0.001**
Age, years				
20–34	Reference		Reference	
35–49	**1.43 (1.32–1.55)**	**<0.001**	**1.38 (1.25–1.52)**	**<0.001**
50–66	**1.51 (1.37–1.66)**	**<0.001**	**1.49 (1.32–1.69)**	**<0.001**
Sex				
Female	Reference		Reference	
Male	**0.90 (0.85–0.97)**	**0.007**	**0.91 (0.85–0.98)**	**0.017**
Age at migration, years				
<18	Reference		Reference	
>18	**1.54 (1.41–1.69)**	**<0.001**	**1.27 (1.13–1.43)**	**<0.001**
Length of stay in Finland, years				
3 to 6.99	Reference		Reference	
7 to 11.99	**0.79 (0.71–0.87)**	**<0.001**	0.92 (0.83–1.03)	0.132
12 or more	1.09 (1.00–1.19)	0.054	**1.28 (1.14–1.43)**	**<0.001**

Model I: Crude model.

Model II: Adjusted for age, sex, age at migration and length of stay in Finland.

Bold values represents statistically significant results.

Table 4 presents the results for the MigCOVID sub-sample. The final result for complete vaccine uptake were similar to the total sample for region of origin and age. When compared with economically active population, those who were economically inactive were less likely to have complete vaccine uptake. Compared with those having excellent Finnish/Swedish language skills, those having basic or intermediate language skill were less likely to have complete vaccine uptake. The vaccine uptake was less likely among those who experienced discrimination and psychological distress compared to those who did not experience such.

**TABLE 4:** The association of sociodemographic and health-related factors with complete COVID-19 vaccine uptake in the MigCOVID sub-sample, Odds Ratio (OR) and 95% confidence interval (CI), (Finland, 2022).

Region of origin	Model I (*n* = 3,668)		Model II (*n* = 3,668)		Model III (*n* = 3,668)	
	OR (95% CI)	*p*-value	OR (95% CI)	*p*-value	OR (95% CI)	*p*-value
Europe (excl. Russia, Estonia)/North America/Oceania	Reference		Reference		Reference	
Russia/former Soviet Union	**0.39 (0.31–0.05)**	**<0.001**	**0.37 (0.29–0.48)**	**<0.001**	**0.34 (0.26–0.44)**	**<0.001**
Estonia	**0.35 (0.27–0.45)**	**<0.001**	**0.32 (0.25–0.42)**	**<0.001**	**0.27 (0.20–0.37)**	**<0.001**
Middle East/North Africa	1.13 (0.85–1.50)	0.409	1.25 (0.94–1.67)	0.130	**1.50 (1.08–2.07)**	**0.014**
Africa (excl. North Africa)	**0.32 (0.24–0.42)**	**<0.001**	**0.34 (0.25–0.45)**	**<0.001**	**0.34 (0.24–0.47)**	**<0.001**
Southeast Asia	**3.56 (2.15–5.88)**	**<0.001**	**3.49 (2.09–5.81)**	**<0.001**	**3.57 (2.10–6.07)**	**<0.001**
Asia (excl. Southeast Asia)/Latin America	**2.76 (1.90–4.00)**	**<0.001**	**2.85 (1.95–4.15)**	**<0.001**	**3.00 (2.00–4.49)**	**<0.001**
Age, years						
20–34	Reference		Reference		Reference	
35–49	**1.52 (1.28–1.80)**	**<0.001**	**1.78 (1.43–2.21)**	**<0.001**	**1.78 (1.42–2.25)**	**<0.001**
50–66	1.09 (0.90–1.32)	0.383	**1.54 (1.17–2.02)**	**0.002**	**1.69 (1.26–2.26)**	**<0.001**
Sex						
Female	Reference		Reference		Reference	
Male	0.96 (0.83–1.11)	0.595	0.86 (0.73–1.02)	0.069	0.86 (0.72–1.02)	0.090
Age at migration, years						
<18	Reference		Reference		Reference	
>18	**1.67 (1.38–2.01)**	**<0.001**	1.00 (0.77–1.29)	0.986	0.91 (0.68–1.22)	0.437
Length of stay in Finland, years						
3 to 6.99	Reference		Reference		Reference	
7 to 11.99	0.87 (0.69–1.09)	0.231	1.09 (0.84–1.41)	0.513	1.09 (0.84–1.44)	0.503
12 or more	**0.73 (0.60–0.90)**	**0.004**	0.96 (0.74–1.25)	0.772	0.90 (0.67–1.20)	0.471
Level of education						
Higher level	Reference		Reference		Reference	
Secondary level	**0.73 (0.58–0.93)**	**0.009**	0.77 (0.58–1.02)	0.065	1.14 (0.85–1.53)	0.373
Basic level or less	**0.65 (0.55–0.77)**	**<0.001**	**0.78 (0.65–0.93)**	**0.007**	0.84 (0.69–1.01)	0.067
Economic activity						
Working full-time/part-time	Reference		Reference		Reference	
Student	**0.72 (0.56–0.92)**	**0.008**	**0.67 (0.50–0.89)**	**0.007**	**0.59 (0.44–0.79)**	**<0.001**
Other	**0.72 (0.57–0.90)**	**0.005**	**0.60 (0.47–0.77)**	**<0.001**	**0.83 (0.63–1.11)**	**0.205**
Unemployed	**0.78 (0.63–0.97)**	**0.024**	**0.65 (0.51–0.82)**	**<0.001**	**0.76 (0.60–0.97)**	**0.027**
Finnish/Swedish language skills						
Excellent	Reference		Reference		Reference	
Beginner/intermediate	**1.20 (1.03–1.40)**	**0.019**	**0.79 (0.65–0.97)**	**0.020**	**0.74 (0.60–0.91)**	**0.004**
Perceived discrimination						
No	Reference		Reference		Reference	
Yes	**0.64 (0.53–0.78)**	**<0.001**	**0.56 (0.45–0.71)**	**<0.001**	**0.46 (0.36–0.58)**	**<0.001**
MHI index						
No psychological distress	Reference		Reference		Reference	
Psychological distress	**0.80 (0.66–0.97)**	**0.025**	0.95 (0.77–1.17)	0.646	**0.74 (0.58–0.93)**	**0.009**
Self-rated health						
Fairly good/good	Reference		Reference		Reference	
Average/fairly poor/poor	**1.30 (1.10–1.54)**	**0.003**	1.15 (0.96–1.39)	0.125	1.23 (1.00–1.50)	0.051

Model I: Crude model.

Model II: Adjusted for age, sex, age at migration and length of stay in Finland.

Model III: Adjusted for age, sex, age at migration, length of stay in Finland, level of education, economic activity, language skills, perceived discrimination, psychological distress and self-rated health.

Bold values represents statistically significant results.

## Discussion

The incidence of SARS-CoV-2 infection was the highest among persons of African (excl. North Africa) origin and the Middle East/North African origin. Complete COVID-19 vaccine uptake was highest among persons originating from Southeast Asia and Asia (excl. Southeast Asia)/Latin America. The complete vaccine uptake was lower for persons originating from Africa (excl. North Africa), Russia/former Soviet Union, and Estonia in comparison with persons originating from rest of Europe/North America/Oceania both before and after adjusting for sociodemographic and other variables. Older age, age at migration >18 years, length of stay in Finland 12 or more years were associated with higher complete vaccine uptake whereas, economically inactive groups, experiences of discrimination and psychological distress were associated with lower complete vaccine uptake in our study.

Migrant origin persons in Norway have been recently reported to have a lower COVID-19 vaccine uptake than those who were Norwegian-born with Norwegian-born parents (8). The Norwegian study also observed lower vaccine uptake among those originating from the East-European countries (Latvia, Bulgaria, Romania, Poland and Lithuania) and higher vaccine uptake among persons originating from the Southeast Asian countries (Vietnam, the Philippines, Thailand) in comparison to persons of Norwegian origin. In our study, vaccine uptake for Estonia and Russia/former Soviet Union group was lower compared with other regional groups. Additionally, our findings on high vaccine uptake among persons originating from South Asian countries are also in consistency with findings from Norway. One key difference between our study and the Norwegian study is that, in addition to at least one COVID-19 vaccine, we also considered previous SARS-CoV-2 infection in our definition of complete vaccine uptake, whereas the Norwegian study only included individuals with at least one dose of COVID-19 vaccine. A previous SARS-CoV-2 infection can be considered equivalent to one dose of vaccine in protecting from future COVID-19 infection (20, 21). Considering the observed high incidence of previous SARS-CoV-2 infections, this information had a significant impact on the calculated outcome variable of vaccine uptake.

Overall, 61% of the persons of migrant origin had complete vaccine uptake in Finland by 16 November 2021. Persons originating from Estonia had the lowest proportion of those with complete vaccine uptake in our study. One explanation for this could be that those of Estonian origin might travel to Estonia to get the vaccination. In such cases, data on receipt of these injections would not appear in the National Vaccine Register of Finland. In addition to persons of Estonian origin, persons originating from Russia/former Soviet Union and Africa (excl. North Africa) were also less likely than persons originating from rest of Europe/North America/Oceania to have complete vaccine uptake. Based on available information from Estonia and Russia, 62% of the population in Estonia and 45% of the population in Russia got at least one or more COVID-19 vaccines by November 2021 (23). Hence, it is also possible that lower vaccine uptake among persons of Russian or former Soviet Union and Estonian origin who took part in the current study, may reflect lower vaccine uptake in their countries of origin.

Vaccine hesitancy is one of the factors contributing to COVID-19 vaccine uptake. The WHO Strategic Advisory Group of Experts (SAGE) working group on vaccine hesitancy defines vaccine hesitancy as a delay in acceptance or refusal of vaccination when vaccination services are available (24). A recent systematic review explored access to and acceptance of COVID-19 vaccines in high-income countries by ethnicity and migrant origin (25). Most of the studies included in the review were conducted in the US and the UK. The study reported that there was consistent evidence of elevated levels of COVID-19 vaccine hesitancy among Black/Afro-Caribbean ethnic groups in the US and the UK. Asians in the US had the highest intention rate to get COVID-19 vaccine (81%) compared to other ethnic groups (40%–68%) (25). Lack of confidence, mainly due to mistrust of government and health systems coupled with poor communication were the main barriers to vaccination uptake among persons of Black ethnicity and migrant origin populations (25). Some other factors associated with lower vaccination intentions in the high-income countries were identified, including having no health insurance, unemployment, lower socio-economic position, female gender, younger age, medical mistrust, less confidence in vaccine efficacy, and less trust in pharmaceutical companies producing the vaccines (26–32).

Persons in the older age groups were more likely to have complete vaccine uptake than those in the younger age groups. Lower socioeconomic position is one of the reasons among persons of migrant origin for poorer outcomes including lower COVID-19 vaccine uptake (4). We observed similar results in our study. Compared to those who were working full-time or part-time, all others (students, unemployed and others) had lower complete vaccine uptake. Experiences of discrimination and psychological distress were factors affecting lower complete vaccination uptake in this study. We did not find any studies on the association of experience of discrimination and vaccine uptake among migrant origin persons. However, there is a body of evidence that showed that experiences of discrimination and psychological stress were adversely related to mental health, physical health, including preclinical indicators of disease, health behaviors, utilization of healthcare, and adherence to medical regimens (33–35). Experiences of discrimination during the COVID-19 pandemic were particularly common among persons of East and Southeast Asian, Middle East, and African origin in Finland (2).

Female sex was often found to be associated with lower COVID-19 vaccination uptake in previous studies (7, 30, 36). However, in our study we observed that males were associated with lower vaccine uptake in the total sample but not in the MigCOVID sub-sample. Persons living longer, i.e., length of stay of 12 years or more were more likely to have complete vaccine uptake compared to those living for 3 to <7 years in the total sample but not in the MigCOVID sub-sample. Generally, the longer the person stays in the country, s/he has a greater chance of integrating in the society and the more likely s/he is to have a good knowledge about the health system and services compared to those who recently moved from another country. Intermediate or beginner Finnish/Swedish language skills were also associated with lower vaccine uptake, highlighting the need for accessible multilingual communications. Those not able to follow the official information on vaccines disseminated by health authorities may be more susceptible to seeking information from other, less reliable sources, and therefore may be at a great risk for exposure to misinformation or disinformation.

### Strengths and Limitations

This is the first study to examine sociodemographic and health-related factors associated with complete COVID-19 vaccine uptake among persons of migrant origin in Finland. A significant strength of the current study is availability of register data on previous laboratory-confirmed SAR-COV-2 infection in addition to the number of the COVID-19 vaccine doses, which were used to define our outcome variable, i.e., complete vaccine uptake. Most of the previous studies relied only on the number of COVID-19 vaccine doses to define vaccine uptake. Vaccination status was extracted from the infectious disease register, and Finnish registers, in general, have good validity. A further strength is population-based random sampling design and availability of sociodemographic register data that could be linked with other register data. Additionally, a significant strength is the availability of self-reported health-related data on for the MigCOVID sub-sample, allowing to take into account such important migration-related variables such as language proficiency, experiences of discrimination, self-rated health, and psychological distress, which would not have been available from registers alone.

Some limitations also need to be addressed. Data on the general population was not available for the current study, which would have been helpful in contextualizing the findings. Additionally, information on COVID-19 vaccines administered in other countries was also not available in Finnish registers. Some of the persons who have migrated to Finland from the neighbouring countries, for example, from Russia or the former Soviet Union, Estonia, and the rest of Europe may have taken a vaccine in their country of origin. Some selection bias was observed when comparing the total study sample and the MigCOVID Survey sub-sample (for example, MigCOVID survey samples had higher complete vaccine uptake than those of total study sample, possibly due to the smaller sample size, see Supplementary Table S2). Such selectivity is commonly observed in population-based surveys, and the selectivity was made transparent in the current study, providing more in-depth insights on the findings than if these would have been presented for the sub-sample only.

### Conclusion

This study highlighted differences in complete COVID-19 vaccine uptake by region of origin and identified sociodemographic and health-related factors associated with vaccine uptake. Findings suggest that young adults, those who are not economically active, had intermediate/beginner level Finnish/Swedish language skills, and those who experienced discrimination and psychological distress were less likely to have complete vaccine uptake. Findings of this study are of high relevance when planning future public health campaigns related to COVID-19 and in future pandemics and highlight the importance of addressing social injustices related to experiences of discrimination as an important measure also from the perspective of pandemic response.

## Abbreviations

COVID-19, Corona virus disease; WHO, World Health Organization; SARS-CoV-2, severe acute respiratory syndrome corona virus 2; UK, United Kingdom; FinMonik, The Survey on Wellbeing among Foreign-Born Population; THL, Finnish Institute for Health and Welfare; MigCOVID, Survey on Impact of Coronavirus Epidemic on Wellbeing among Foreign-born Population; MHI, Mental Health Index; OR, Odds Ratio; CI, Confidence Interval; SAGE, The WHO Strategic Advisory Group of Experts; US, United States.
