# Supplementary material for: Incidence of SARS-CoV-2 Infection and Factors Associated With Complete COVID-19 Vaccine Uptake Among Migrant Origin Persons in Finland
Source: Int J Public Health. 2023 May 3;68:1605547. doi: 10.3389/ijph.2023.1605547 (PMC10189547; doi:10.3389/ijph.2023.1605547)
Supplement: Supplementary file 1 [file DataSheet1.docx]

Supplementary Table 1: Composition of the migrant groups by country of origin.

| **Groups** | **country of origin** |
| --- | --- |
| Russia and the former Soviet Union | Former Soviet Union, Russia |
| Estonia | Estonia |
| Europe (excl. Russia, Estonia), North America, Oceania | Poland, United Kingdom, Germany, Former Yugoslavia, United States, Hungary, Spain, Sweden, The Netherlands, France, Ukraine, Bulgaria, Romania, Latvia, Italy, Lithuania, Greece, Canada, Portugal, Switzerland, Denmark, Australia, Belgium, Ireland, Albania, Former Czechoslovakia, Austria, Slovakia, Belarus, Former East Germany, Bosnia and Herzegovina, Czech Republic, Iceland, Malta, New Zealand, North Macedonia, Serbia, Serbia and Montenegro, Slovenia |
| Middle East and North Africa | Afghanistan, Iraq, Iran, Turkey, Syria, Morocco, Egypt, Sudan, Israel, Lebanon, Tunis, Algeria, Arab Emirates, Armenia, Georgia, Jordan, Libya, Saudi Arabia, Yemen |
| Africa (excl. North Africa) | Somalia, Nigeria, Ethiopia, Kenya, Ghana, Democratic Republic of the Congo, Tanzania, Gambia, Angola, Cameroon, Guinea, Ivory Coast, Liberia, Mauritania, Rwanda, Senegal, South Africa, South Sudan, Uganda, Zambia, Zimbabwe |
| Southeast Asia | Thailand, Vietnam, Philippines, Myanmar, Indonesia, Cambodia, Malaysia, Singapore |
| Asia (excl. Southeast Asia)/Latin America | China, Japan, Taiwan, South Korea, Hong Kong, India, Nepal, Bangladesh, Pakistan, Kazakhstan, Sri Lanka, Kyrgyzstan, Uzbekistan, Brazil, Peru, Mexico, Cuba, Chile, Colombia, Argentina, Venezuela, Ecuador, Barbados, Bolivia, Costa Rica, Dominican Republic, Guyana, Haiti, Honduras, Jamaica, Nicaragua, Uruguay |

Supplementary Table 2: Incidence of laboratory confirmed SARS-CoV-2 infection and COVID-19 vaccine uptake in the MigCOVID sub-sample.

| **Migrant groups** | **SARS-CoV-2 infection** | **COVID-19 Vaccine dose** | | | | | **complete vaccine**  **uptake*** |
| --- | --- | --- | --- | --- | --- | --- | --- |
|  |  | | **0** | **1** | **2** | **3** |  |
|  | **% (95% CI)** | | **% (95% CI)** | **% (95% CI)** | **% (95% CI)** | **% (95% CI)** | **% (95% CI)** |
| Russia/former Soviet Union (n=797) | 7.6 (5.2–10.9) | | 32.8 (27.8–38.2) | 6.6 (4.5–9.7) | 58.0 (52.5–63.3) | 2.6 (1.2–5.4) | 61.5 (56.1–66.7) |
| Estonia, (n=498) | 13.6 (8.1–22) | | 37.6 (29.1–46.9) | 5.9 (2.6–13) | 54.6 (45.4–63.6) | 1.9 (1.0–3.5) | 58.7 (49.4–67.4) |
| Europe (excl. Russia, Estonia) /North America/Oceania, (n=691) | 4.4 (2.5–7.6) | | 14.3 (10.1–19.8) | 5.6 (2.9–10.6) | 79.1 (72.7–84.2) | 1.0 (0.4–2.4) | 80.3 (73.9–85.4) |
| Middle East/ North Africa, (n=572) | 24.4 (17.9–32.4) | | 17.0 (11.9–23.6) | 4.7 (2.4–9.1) | 77.8 (70.8–83.5) | 0.6 (0.2–1.7) | 82.1 (75.5–87.2) |
| Africa (excl. North Africa), (n=335) | 23.4 (15.2–34.1) | | 33.5 (24.2–44.2) | 11.8 (6.4–20.8) | 47.8 (37.3–58.4) | 6.9 (2.6–17.2) | 56.4 (45.6–66.6) |
| Southeast Asia, (n=289) | 4.2 (1.7–9.9) | | 2.8 (1.2–6.2) | 5.6 (2.5–12) | 89.0 (82.1–93.5) | 2.6 (0.9–7.6) | 93.5 (88.6–96.4) |
| Asia (excl. Southeast Asia) /Latin America, (n=487) | 7.9 (3.7–16.2) | | 7.1 (4.2–11.7) | 2.0 (1.1–3.8) | 90.0 (85.3–93.3) | 0.9 (0.5–1.8) | 91.8 (87.2–94.9) |
| Total, (n=3,668) | 11.6 (9.7–13.9) | | 21.8 (19.4–24.4) | 5.8 (4.5–7.4) | 70.4 (67.5–73) | 2.1 (1.4–3.1) | 73.9 (71.2–76.5) |

*****Complete vaccine uptake implies if a person had a previous SARS-Cov-2 infection and one dose vaccination or if person has two or more vaccines doses and no previous SARS-Cov-2 infection.
